# Red Sulphur Springs

**Published:** 1840-10

**Authors:** 


*Red Sulphur Springs.*—I passed on without stopping long at any of the springs until I reached the Red Sulphur, which is the most distant of them. It is situated in the county of Munroe, seventeen miles from the Salt Sulphur, and of course forty-two miles from the White. The spring is concealed in a glen in the midst of hills, which surround it completely, forming, however, a narrow valley, the length of which is much greater than the breadth. These hills were at one time covered with high trees; but the proprietor of the spring, Mr. Burke, has cleared them away, from a well-grounded apprehension on the part of the invalids that they obstructed the circulation of the air, and rendered the place damp. The hills shelter the buildings and the whole inclosure from the cutting winds which are often felt in the springs near the Allegheny mountains; the climate therefore is extremely mild in the summer season, and subject only to one inconvenience—the damp fogs and occasional chillness in the evening; this is, however, readily guarded against by a little fire in the cabins. Most of the invalids avoid walking after sunset, or in the early part of the morning until the dew is cleared away.

The summer climate is therefore not unexceptionable; but is still vastly better for a pulmonary invalid than the excessive heat of the eastern plains, or the cold breezes of the sea-side. In pulmonary cases, properly speaking, I have observed no inconvenience; but I have met with asthmatics who were injured quite seriously by a journey to the mountains, which was incautiously advised by their medical attendant. The sharp air and the alternations of temperature produce much greater effects upon them than on those who are disposed to tuberculous diseases.

The reputation of the Red Sulphur Springs is chiefly founded upon its efficacy in either alleviating or curing pulmonary diseases, including catarrhs of long standing, and commencing tuberculous cases. Even in patients who have long labored under the latter form of disease, the benefits of the spring have been much sought for. A reputation of this kind, which arose gradually, and was sustained for many years, notwithstanding many disadvantages of situation, and an almost absolute dearth of comfortable accommodations for the sick, which then existed, cannot be unfounded. It is true that an exaggerated estimate of the powers of the water, and a carelessness in the selection of patients who have been from time to time sent to the spring, have produced an impression which is unfavorable to its true value.

I met with a remarkable instance of this. On my return from the Red Sulphur, at the Salt Sulphur I found a young married lady who had just arrived from a long journey of sixteen days; during this whole period she had labored under a colliquative diarrhœa, and an exhausting hectic, with the thick yellow expectoration which indicates the rapid softening of tuberculous matter. I was then called to see her in consultation with a gentleman from the same part of the country in which she resided, and found her rapidly sinking; at our earnest request, the friends of the lady, who were utterly unconscious of her danger, consented to give up their intention of proceeding farther; she died the next day, and but for our interference, would have sunk in the course of the last day's journey. These extreme cases are not frequent, but there are many instances in which much mischief arises from advising patients in the last stage of pulmonary phthisis to resort to the Red Sulphur, precisely as in similar circumstances it is pernicious and morally wrong to direct them to abandon the comforts of home in the vain hope of seeking for health in a warmer climate. No patients who are exhausted by hectic fever, and copious purulent expectoration proceeding from cavities, should be sent to a distant place; and a long journey should be still more sedulously avoided if there is much diarrhœa. The latter symptom is generally aggravated by the journey, and the patient is besides unable to take the Red Sulphur water in sufficient quantities to produce any sensible effects; for the quantity, ra- than the quality of the water, tends to increase the discharge from the bowels. It is under certain circumstances desirable for invalids in the last stages of pulmonary disease to leave their accustomed air, and seek for a restoration of strength, if not an entire recovery; but these persons should seek a nearer retreat, and one exempt from the fatigues of a mountain journey.

The explanations which are given of the mode in which the water of the Red Sulphur Spring acts in relieving phthisis, do not seem to me to be well founded. It is thought that it has a direct power of diminishing the frequency of the pulse. It is true that this result does follow in a certain proportion of cases; but on a careful examination of many patients who took the water, I found that this effect did not occur at the beginning of many of the cases. It is true that when the irritation of the disease was to a certain extent subdued, the pulse became less frequent; but this did not appear to result from any peculiar action of the water. Its first action was that of a sedative, perhaps more nearly resembling minute doses of hydrocyanic acid than any thing else. It caused a tendency to sleep in nearly every patient, and in some gave rise to considerable headache, and a disagreeable sensation of fulness, which lasted for a few days. The water passed off by the kidneys, producing little effect on the bowels or the skin. It may, however, be readily determined to the surface by proper attention to warmth.

The quantity of water taken is very various; in former times it was much more freely drank than at present; invalids now rarely take more than eight tumblers, about three pints, in a day. But if the water does not produce a permanently disagreeable effect upon the alimentary canal, it may be properly taken in much larger doses, say from ten to twenty glasses of the kind usually employed at the spring. But while taking the water the invalid should take constant and regular exercise; this precaution is one always to be observed when using mineral waters, but certainly by no class of patients more faithfully than those disposed to phthisis. Yet simple and obvious as is this rule of hygiene, the patients have of late been singularly negligent in this matter, and often limit their exercises to an occasional game at ninepins, and a quiet stroll about the grounds. The water should be taken in divided quantities before each meal, but especially before breakfast. Thus, if the allowance be eight glasses, three of them should be taken before breakfast, in the space of an hour or two; two before dinner, allowing the same period for it; one before supper, and two in the evening. But between every glass, as much exercise as practicable without producing fatigue, should be taken if the weather be fine. If the morning should be cool, it is proper either to exercise within doors, or in the piazzas, which, at the Red Sulphur, extend several hundred feet in length.

Much of the benefit of mineral water depends upon the facility with which it is absorbed, and, as it were diffused throughout the system: this diffusion is greatly promoted by vigorous exercise, which prevents the unpleasant sensation of a load or fulness at the stomach, that often occurs under different circumstances, Exercise is more necessary while taking a water like that of Red Sulphur, which contains but a very small proportion of saline matter, and depends for its action chiefly upon its gaseous elements, which are less readily determined towards a particular organ.

The red deposit which gives name to the spring is less copious this year than usual; in fact, it is scarcely a deposit; a portion of sulphur is combined with a peculiar organic substance, apparently some variety of the cryptogamous plants. The spring has been carefully cleared out, and the sides for the deposition of the plant may have been removed. Some of the old visiters to the spring fancy that the strength of its waters has been impaired by the copious rains of the summer, but this opinion does not obtain amongst others.

The action of the water is certainly that of a mild alterative, as well as sedative, and it is useful not merely in cases of commencing phthisis, but of many sub-acute inflammations of the mucous membranes. Indeed, in pulmonary disease the water is apparently most beneficial in the stages of the diseases in which there is a slight irritation, sufficient to cause some feverishness, but not to give rise to very acute symptoms. The acute forms of phthisis are usually aggravated by a residence at the Red Sulphur; at least this was my experience during the present summer; but this aggravation manifestly depends more on the irritation of the journey and peculiarities of climate, than on any action of the water. The therapeutic rule, that much exercise, or excitement of any kind, is injurious in acute phthisis, always holds good.

The disorders of the mucous membranes in which this water is the most useful, are chronic inflammations of the stomach and bowels, especially the varieties which are sub-acute from the beginning: when the case is one of chronic dysentery following the acute form of the disease, the good effects of the water seem to me more problematical; but of this I have no personal experience. In chronic irritation of the urinary passages a like good effect has often been noticed; I recommended it in cases of this kind, but was not able from my own experience to confirm the results which have been previously observed. In these diseases the water acts medicinally by its alterative virtues; in the functional diseases of the heart the action of the water is more complex, and is partly derived from its alterative, and partly from its sedative qualities. An interesting case of this kind occurred to me: a young gentleman, a student of the University of Virginia, was affected with a disease of the heart, chiefly symptomatic, and dependent upon nervous derangement and dyspepsia, but in part connected with a real enlargement of this organ. After remaining some time at the White Sulphur without benefit, he resorted to the Red Sulphur,—and after taking the waters for a few days with the usual precautions, his symptoms rapidly declined. A single case of this kind is of no value, but the general experience is decidly favorable to the effects of the water in this form of disease.

A mild mineral water like the Red Sulphur, which contains, it is believed, but little more active ingredients than sulphuretted hydrogen and nitrogen gas, must derive much of its virtue from the temperature and quantity of the water. The medicinal ingredients act probably quite as effectually by the direction which they give to the water, as by the remedial properties peculiar to themselves.—*Medical Examiner, Aug. 15th,* 1840.

